# Spontaneous rupture of a giant thymoma causing hemothorax and cardiopulmonary collapse

**DOI:** 10.1016/j.rmcr.2026.102430

**Published:** 2026-05-01

**Authors:** Swetha Lakshminarayanan, Miguel A. Ureña-Perez, Israel Ramirez-Sanchez, Claire Bensard, Patricia A. Thistlethwaite

**Affiliations:** a Division of Cardiothoracic Surgery, University of California, San Diego, United States; b National Polytechnic Institute, Higher Education School of Medicine Graduate Studies and Research, Mexico City, Mexico

**Keywords:** Thymoma, Hemothorax, Spontaneous rupture, Cardiopulmonary collapse

## Abstract

Spontaneous rupture of a thymoma is a rare event. An 81-year-old female presented with an asymptomatic 14-cm thymoma in the mid-upper mediastinum, extending to the right diaphragm for thymectomy. Two days before surgery, she developed acute dyspnea and chest pain. Admission chest roentgenogram showed a new large right pleural effusion. Thoracoscopic drainage of 2200 mL of blood mixed with necrotic tumor debris revealed a posterior rupture of her thymoma. The mass was resected via median sternotomy, and the chest irrigated. Her postoperative course was uneventful. Final pathology showed Type A thymoma with an 8-cm perforation, with ruptured intratumoral and capsular veins.

## Highlights

• Spontaneous rupture of giant thymoma may cause clinical deterioration due to hemodynamic instability and pulmonary failure.
• Thymoma rupture is associated with hemothorax from ruptured capsular and intratumoral veins.
• Prompt recognition and surgical intervention for spontaneous rupture of a thymoma is life-saving.

## 1 Introduction

Although thymomas are the most common anterior mediastinal mass in adults, they are uncommon neoplasms, with an estimated incidence of 0.13 - 0.57 per 100,000 person-years [1]. In non-myasthenic patients, the majority of cases are detected incidentally on chest imaging. Some individuals present with symptoms of pain, cough, or chest tightness. Approximately 35 - 45% of thymoma patients manifest symptoms of the paraneoplastic syndrome of myasthenia gravis [2]. Spontaneous rupture of a thymoma, resulting in hemothorax, mediastinal hemorrhage, or intraparenchymal pulmonary hemorrhage are extremely rare events. We report a case of spontaneous rupture of a giant thymoma, resulting in dyspnea and shock managed by emergency resection via median sternotomy.

## 2 Case presentation

**Fig. 1:**
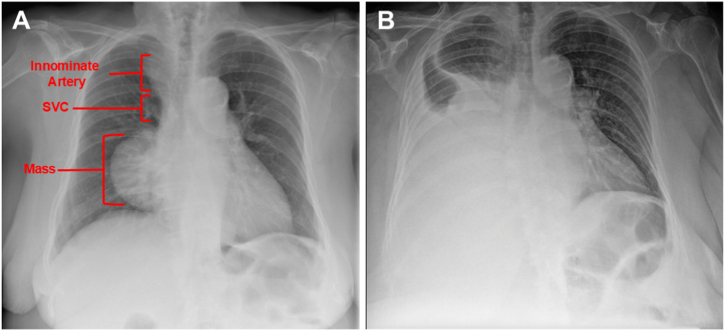
Radiographic findings of ruptured thymoma. Chest roentgenogram, posterior-anterior (PA) view obtained one week prior to admission (A) and chest roentgenogram, anterior-posterior (AP) view on the day of surgery (B), showing a new right pleural effusion with mild mediastinal shift to the right.

**Fig. 2:**
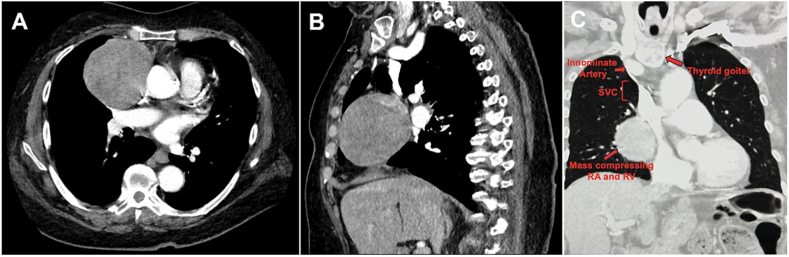
Chest computed tomography imaging with IV contrast. Axial view (A) demonstrating a heterogeneous mass in the mediastinum, and sagittal view (B) showing extension of the mass to the right diaphragm. Coronal view (C) demonstrating location of small substernal goiter in relation to the mass. Structures are labelled in red.

An asymptomatic 81-year-old, 132-cm tall Hispanic woman was found to have a large mediastinal mass on posterior-anterior (PA) chest roentgenogram (CXR) (Fig. 1A). Follow-up chest computed tomography (CT) with IV contrast demonstrated a 14-cm non-calcified mass in the mid-upper mediastinum, draping over the right hilum, and extending to the diaphragm (Fig. 2A and B) and a 3-cm substernal goiter (Fig. 2C). Percutaneous biopsy of the 14-cm non-calcified mass via an anterior chest wall approach was obtained at an outside hospital, revealing thymoma of unspecified type. She was scheduled for elective excision. Two days prior to her operation, she developed acute shortness of breath and chest pain after a coughing episode. She presented for surgery, carried in by her sons, with severe dyspnea, diaphoresis, cool extremities, and confusion. Oxygen saturations and blood pressure were recorded as 72 - 82% on 100% FiO_2_ non-rebreather face mask and 70/30 mmHg, respectively. Anterior-posterior (AP) CXR showed a large right pleural effusion, with mild mediastinal shift to the right (Fig. 1B). A blood sample demonstrated new anemia (hemoglobin: 4.0 g/dL), compared to her preoperative hemoglobin of 11.1 g/dL, measured one week prior. She had no response to rapid fluid resuscitation, instituted before blood tests came back, in the preoperative holding area. She was taken emergently to the operating room.

**Fig. 3:**
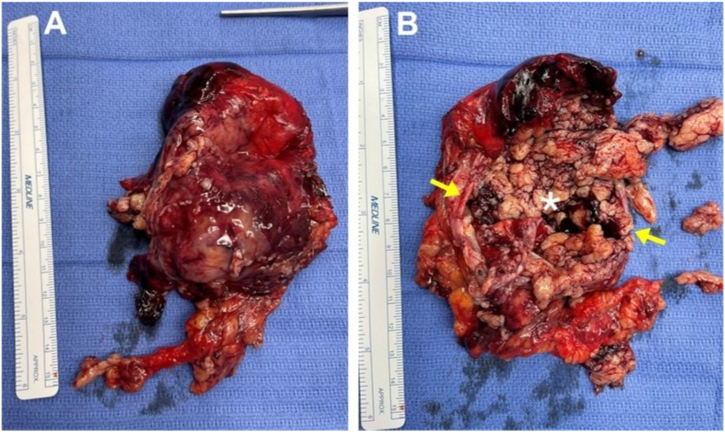
Gross pathology specimen of the resected thymoma. Anterior view (A) showing the intact encapsulated mass. Posterior view (B) demonstrating rupture of capsular surface (yellow arrows), with necrotic tumor center (∗).

The patient was intubated awake and had a bronchial blocker placed in the right mainstem bronchus for lung isolation. Video-assisted thoracoscopic drainage of 2200 mL of blood mixed with yellow tissue debris was performed, revealing a large mass that extended from the mid portion of the superior vena cava to the diaphragm, draping anterior to the right hilum, with an 8-cm rupture on the posterior surface (Fig. 3). The lesion was easily separated from the right lung, but was adherent to the right pulmonary artery and mediastinal pleura. The patient was then placed in the supine position and median sternotomy was performed. Her mass was resected en bloc with 6-cm of pericardium, sparing the right phrenic nerve, after gaining intrapericardial control of the right hilum as well as the azygous-superior vena caval confluence. The right chest and mediastinum were irrigated copiously with retrieval of multiple pieces of yellowish necrotic tissue. The patient had an uneventful postoperative course and was discharged on the seventh postoperative day. The pathology report classified her tumor as a 14-cm Type A thymoma, with necrosis involving 40% of the tumor mass, without direct invasion into the mediastinal pleura, mediastinal fat, or continguous normal thymic tissue. All 16 lymph nodes resected were negative. Her surveillance chest CT, performed six months after surgery, showed no evidence of drop metastases.

## 3 Discussion

Spontaneous rupture of a thymoma causing hemothorax, hemomediastinum, or intraparenchymal pulmonary bleeding is an extremely uncommon occurrence, with few cases reported. Our case contributes to this limited cohort of cases and highlights a life-threatening but potentially reversible complication of thymoma. Thymoma rupture is associated with acute clinical onset, often with dyspnea and hemodynamic instability [3]. In our case, clinical deterioration was likely triggered by an 8-cm tear on the posterior aspect of the thymoma, with actively bleeding capsular and intratumoral veins. The hypotension seen in this patient was likely due to two mechanisms. First, she experienced Stage 4 shock, defined as acute loss of more than 40% of blood volume, leading to poor tissue perfusion, rapid heart rate, and decrease in blood pressure [4]. Second, the inability of the patient to adequately ventilate the right lung, due to the sudden space-occupying effect of more than 2 L of blood in the right pleural space may also have contributed to hypoxemia, causing poor cardiac perfusion, worsening cardiac function, and resultant hypotension.

The cause of spontaneous rupture of a thymoma is unknown. Two distinct pathologies have been described, including small focal transmural rupture of a vein on the outer capsule surface or full-thickness capsular rupture with intratumoral and capsular surface bleeding [5,6]. Several mechanisms have been proposed based on 11 published case reports [7], detailing these entities. These include: rapid expansion in tumor growth causing capsular and/or vascular rupture, intratumoral hemorrhage that increases pressure within the tumor leading to capsular thinning and rupture, acute increase in intrathoracic pressure from coughing or Valsalva maneuvers, and sudden surges of blood pressure. These mechanisms may exist independently or synergistically to trigger rupture. Tumor rupture has not been reported in individuals with hereditary or autoimmune vasculitides. Interestingly, the risk of spontaneous rupture of a thymoma has not been correlated with specific thymic histology or tumor size, with lesions from 7 mm to 14.5 cm reported [8,9]_._

Secondary effects of mediastinal shift, mediastinal compression, and acute blood loss are usually associated with hemodynamic instability, leading to cardiovascular collapse. Either misdiagnosis or delay in diagnosis may hinder definitive treatment for this life-threatening condition.

## 4 Conclusion

In conclusion, spontaneous rupture of a thymoma may be a life-threatening event, necessitating emergency surgical intervention. This case underscores the importance of having a high index of suspicion for tumor rupture in cases of spontaneous hemothorax with an associated thymoma. Favorable outcome is possible with prompt diagnosis and intervention. Serial follow-up imaging every three months for two years, followed by every six months up to five years, then annually for this patient has been instituted, due to the concern for the development of drop metastases from tumor dissemination when the thymoma ruptured.

## CRediT authorship contribution statement

**Swetha Lakshminarayanan:** Writing – original draft, Visualization, Validation. **Miguel A. Ureña-Perez:** Writing – review & editing, Visualization, Validation. **Israel Ramirez-Sanchez:** Writing – review & editing, Validation. **Claire Bensard:** Validation, Resources. **Patricia A. Thistlethwaite:** Writing – original draft, Validation, Supervision, Resources, Project administration, Funding acquisition, Conceptualization.

## Ethical approval

Ethics approval was not required in accordance with our institutional guidelines.

## Consent

Informed consent was obtained from the patient for publication of this case report and accompanying images.

## Data availability statement

Not applicable.

## Funding sources

This work was supported by the 10.13039/100000002National Institutes of Health grant: NIH/NLHBI R01 HL169861.

## Declaration of competing interest

The authors declare that they have no known competing financial interests or personal relationships that could have appeared to influence the work reported in this paper.
